# Burden of Chikungunya Virus Infection during an Outbreak in Myanmar

**DOI:** 10.3390/v15081734

**Published:** 2023-08-14

**Authors:** Mya Myat Ngwe Tun, Aung Kyaw Kyaw, Khine Mya Nwe, Su Su Myaing, Ye Thu Win, Shingo Inoue, Yuki Takamatsu, Takeshi Urano, Hlaing Myat Thu, Saw Wutt Hmone, Kyaw Zin Thant, Kouichi Morita

**Affiliations:** 1 Department of Tropical Viral Vaccine Development, Institute of Tropical Medicine, Nagasaki University, Nagasaki 852-8523, Japan; moritak@nagasaki-u.ac.jp; 2 Department of Virology, Institute of Tropical Medicine, Nagasaki University, Nagasaki 852-8523, Japan; drkhinemyanwe@gmail.com (K.M.N.); yukiti@nagasaki-u.ac.jp (Y.T.); 3 Center for Vaccines and Therapeutic Antibodies for Emerging Infectious Diseases, Shimane University, Izumo 690-8504, Japan; turano@med.shimane-u.ac.jp; 4 Department of Medical Research, Ministry of Health, Yangon 11191, Myanmar; akkyawdmr@gmail.com (A.K.K.); susumyaing.pol@gmail.com (S.S.M.); hmyatthu28@gmail.com (H.M.T.); 5 550-Bedded Children Hospital (Mandalay), Department of Medical Services, Ministry of Health, Mandalay City 05021, Myanmar; dryethuwin@gmail.com; 6 Kenya Research Station, Institute of Tropical Medicine, Nagasaki University, Nagasaki 852-8523, Japan; pampanga@nagasaki-u.ac.jp; 7 Department of Pathology, University of Medicine-1, Ministry of Health, Yangon 11131, Myanmar; sawwuthmone@gmail.com; 8 Myanmar Academy of Medical Science, Yangon 11201, Myanmar; drkz.thant@gmail.com; 9 DEJIMA Infectious Disease Research Alliance, Nagasaki University, Nagasaki 852-8523, Japan

**Keywords:** molecular detection, seropositivity, chikungunya burden, Myanmar

## Abstract

Chikungunya virus (CHIKV) infection is a re-emerging arboviral disease with no approved vaccine, although numerous options are in development. Before vaccine implementation, disease burden, affected age group, and hospitalization rate information should be documented. In 2019, a sizeable outbreak of the East Central South African genotype of CHIKV occurred in Myanmar, and during this period, a cross-sectional study was conducted in two regions, Mandalay and Yangon, to examine the molecular and seropositivity rate of the CHIKV infection. The participants (1124) included dengue-suspected pediatric patients, blood donors, and healthy volunteers, who were assessed using molecular assays (quantitative real-time RT-PCR), serological tests (anti-CHIKV IgM capture and IgG indirect enzyme-linked immunosorbent assays), and neutralization tests. The tests confirmed the following positivity rates: 11.3% (127/1124) for the molecular assay, 12.4% (139/1124) for the anti-CHIKV IgM Ab, 44.5% (500/1124) for the anti-CHIKV IgG Ab, and 46.3% (520/1124) for the CHIKV neutralizing Ab. The highest rate for the molecular test occurred with the dengue-suspected pediatric patients. The seroprevalence rate through natural infection was higher in the healthy volunteers and blood donors than that in the pediatric patients. The results of this study will help stakeholders determine the criteria for choosing appropriate recipients when a CHIKV vaccine is introduced in Myanmar.

## 1. Introduction

Chikungunya virus (CHIKV) is a virulent emerging disease that constitutes one of the 29 recognized species in the *Alphavirus* genus of the Togaviridae family [1]. CHIKV is mainly transmitted through *Aedes aegypti* mosquito bites, although after the 2010 outbreak, the E1:A226V mutation transferred the virus to the *Aedes albopictus* mosquito population, which caused CHIKV to spread to Europe and the rest of the world [2,3,4]. CHIKV was first detected in Asia in 1954, and it has continued to circulate in South Asia and South East Asia countries. The first case of CHIKV infection in Myanmar was serologically confirmed in 1976, and the first isolate of the East Central South African (ECSA) genotype was identified during the 2010 outbreak [5,6]. Subsequently, CHIKV silently circulated in the region, and 9 years after the initial outbreak, a new clade of the ECSA genotype emerged that caused a large outbreak in Myanmar [7,8].

CHIKV infection has clinical manifestations that range from asymptomatic to fatal. The clinical manifestations of dengue virus (DENV) and CHIKV infections are similar such as high fever, headache, nausea, vomiting, rashes, arthralgia, and myalgia; however, acute arthritis and rash are more prominent in CHIKV infection [8,9,10]. In addition, atypical manifestations and neurological manifestations of CHIKV have been reported [11]. Symptomatic cases can find out by passive surveillance in hospital settings. On the other hand, active surveillance in the community can identify both symptomatic and sub-clinical asymptomatic cases [12]. Serology can be used to determine the extent of the disease since CHIKV infection can present as asymptomatic, and this method measures antibodies (Ab) produced after exposure or vaccination against the specific pathogen [13]. Using serology-based methods, the level of exposure of the specific pathogen can be determined by measuring both specific and neutralizing antibodies. A variety of methods have been used to identify recent or past infections depending on the stages of symptomatic patients. Virus isolation or molecular techniques are the superior methods for acute infection diagnosis [14]. Anti-CHIKV IgM Ab is a specific indicator of a recent infection, as it usually appears one week after exposure to the virus and can persist for three to 18 months after infection [15,16]. In addition, anti-CHIKV IgG Ab is used to determine recent or past infections.

According to the World Health Organization, CHIKV infection has become a major public health concern globally with considerable socio-economic challenges [3,4]. To reduce the burden of diseases and socio-economic challenges, vaccination is an effective way to prevent the disease. Although there is currently no licensed vaccine for CHIKV, many candidates are in development [17]. In anticipation of a vaccine, the burden of the outbreak and immune status against the CHIKV infection were assessed [18], using both active and passive surveillance during the 2019 outbreak in Myanmar. Therefore, this study aimed to identify the proportion of dengue-suspected pediatric patients, blood donors, and healthy volunteers who achieved immunity through recent CHIKV infection or during the 2019 outbreak in the Mandalay and Yangon study areas.

## 2. Materials and Methods

### 2.1. Participant Recruitment and Study Areas

The study population comprised 1124 participants and serum samples collected during 2019. These included 196 clinician-diagnosed dengue-suspected pediatric patients admitted to the Mandalay Children Hospital, 691 blood donors from the blood bank of the Mandalay General Hospital, and 237 apparently healthy persons (healthy volunteers) during periodic medical examinations from Yangon private clinics with no history of fever or joint pains within one week of fulfilling the criteria for blood donation. For this study, participants from Yangon (the largest city) and Mandalay (the second largest city) in Myanmar were selected. At the recruitment of study participants, the investigators followed the standard operating procedures and we informed the participants and their clinicians immediately if the participants had positive results on their blood tests.

### 2.2. Molecular Detection of the CHIKV Genome Using Quantitative Reverse Transcription Polymerase Chain Reaction (qRT-PCR)

To detect the CHIKV genome, RNA extraction from serum samples was conducted using the Qiagen Viral RNA Extraction Kit (Qiagen, Hilden, Germany). Real-time cDNA synthesis (RT) was performed using 2 μL of 5 × PrimeScript RT Master Mix, 500 ng of RNA template, and RNase-free water up to 10 μL (Takara, Shiga, Japan), and thermal cycling was conducted at 37 °C for 15 min, followed by 85 °C for 15 min. The TB Green real-time PCR reaction mixture (Takara, Shiga, Japan) contained 5 μL of TB Green Premix Ex Taq II, 0.2 μL of ROX reference Dye I, 1 μL of cDNA, 3 μL of RNase free water, and 0.4 μL each of the 10 μM forward (NSP2-GGCAGTGGTCCCAGATAATTCAAG) and reverse (NSP2-GCTGTCTAGATCCACCCCATACATG) primers, which have been described in previous studies [19]. PCR amplification was conducted at 95 °C for 30 s followed by 40 amplification cycles of 95 °C for 5 s and 60 °C for 34 s [19]. Cycle threshold (Ct)value-37 was used as the cutoff point for determining positive cases of CHIKV in this study.

### 2.3. Detection of Anti-CHIKV IgM and IgG Antibodies

To identify recent CHIKV infections during this outbreak, anti-CHIKV IgM antibody (Ab) levels were measured from the serum samples of the dengue-suspected pediatric patients, blood donors, and healthy volunteers from the two study areas. An in-house IgM capture enzyme-linked immunosorbent assay (ELISA) that was validated in a previous study was performed according to the previously described procedures [20,21,22] The sensitivity of the in-house anti-CHIKV IgM capture ELISA was 98.3% (95% CI: 90.9–100%) and specificity was 88.0% (95% CI: 71.8–96.6%), with an accuracy of 94.6% [6,7,20,21,22]. The optical density (OD) was read, and each positive control or sample OD was divided by the OD of the negative control; a P/N ratio ≥ 2 was considered positive.

To identify previous exposures to or immunities against CHIKV infections in dengue-suspected pediatric patients, blood donors, and healthy volunteers, anti-CHIKV IgG Ab presence was determined using indirect IgG ELISA according to procedures described in a previous study [20,22,23]. The IgG titers of patient sera were determined from a positive standard curve, and a titer ≥ 3000 was considered IgG-positive. The sensitivity of the in-house anti-CHIKV IgG indirect ELISA was 94.2% (95% CI: 88.9–97.5%) and specificity was 100% (97.8–100%), with an accuracy of 97.4% [6,7,20,22,23].

### 2.4. Neutralization Assay for the CHIKV Virus

The anti-CHIKV-IgM and anti-CHIKV IgG Ab positive samples were analyzed for the neutralizing antibody against CHIKV using the 50% focus reduction neutralization test (FRNT_50_) as described in previous studies [6,20,23]. First, the serum samples were diluted with 2% fetal calf serum minimum essential medium (FCS MEM) beginning with a 1:80 ratio that was heated at 56 °C for 30 min. The heat-treated samples were then mixed with equal volumes of 40 focus-forming units of CHIKV and incubated at 37 °C for 1 h, after which the serum and virus mixtures were transferred into 96-well plates of confluent Vero cell monolayers and incubated at 37 °C for 1.5 h. Subsequently, the cells were overlaid with 150 μL of 2% FCS MEM containing 1% methylcellulose 4000 (WAKO Pure Chemical Industries, Osaka, Japan). The plates were incubated at 37 °C with 5% CO_2_ for 36 h, after which time the cells were fixed, blocked, and permeabilized following the methods of previous studies. Viral foci were detected by immunostaining the cells with C57BL/6J mouse anti-CHIKV serum, peroxidase-conjugated anti-mouse IgG (American Qualex, San Clemente, CA, USA), and DAB substrate (WAKO Pure Chemical Industries, Osaka, Japan). The endpoint serum dilution that produced a ≥50% reduction over the mean number of the control well was considered the FRNT_50_ titer, and IgG- or IgM-positive samples with a neutralizing titer of ≥10 were classified as CHIKV infected [6,20,23].

### 2.5. Statistical Analysis

Microsoft Excel was used for data entry and analysis was performed using GraphPad Prism (10.0) software. The test positivity rates were described using numbers and percentages, while IgG Ab titers were identified using medians (interquartile range). The Kruskal–Wallis and Mann–Whitney tests were used to determine the difference in the medians among the groups. A *p*-value of less than 0.05 was used as the cut-off point for statistical significance.

## 3. Results

### 3.1. Demographic Data of the Participants

The study participants consisted of 196 clinically diagnosed dengue-suspected pediatric patients (<13 years of age) from the 550-bed Mandalay Children Hospital, 691 blood donors (>18 years of age) from the blood bank of the Mandalay General Hospital, and 237 healthy volunteers (>18 years of age) during periodic medical examinations from private clinics in the Yangon Region. The demographic data of the participants are shown in Table 1.

### 3.2. Proportion of Positive Molecular Tests among Study Population

**Figure 1:**
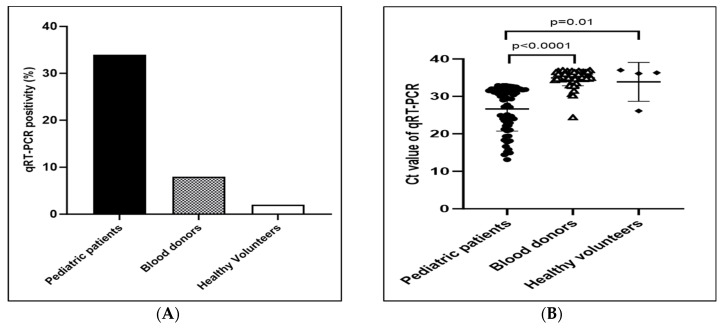
The positivity rate for qRT-PCR positivity (**A**) and Ct value of qRT-PCR (**B**) in different health statuses.

Of the 1124 participants, 127/1124 (11.3%) returned positive molecular tests. The viral RNA by qRT-PCR test detected CHIKV in 66/196 (33.7%) of the dengue-suspected pediatric patients, 57/691 (8.2%) of the blood donors, and 4/237 (1.7%) of the healthy volunteers. The detection of qRT-PCR positivity rate was the highest in the dengue-suspected pediatric patients, followed by the blood donors and healthy volunteers (Figure 1A). The mean Ct value of the viral RNA genome in dengue-suspected pediatric patients was significantly lower than in blood donors and healthy volunteers (Figure 1B).

### 3.3. Immune Status (Anti-CHIKV IgM and Anti-CHIKV IgG Abs Positivity) against CHIKV among Study Population

**Figure 2:**
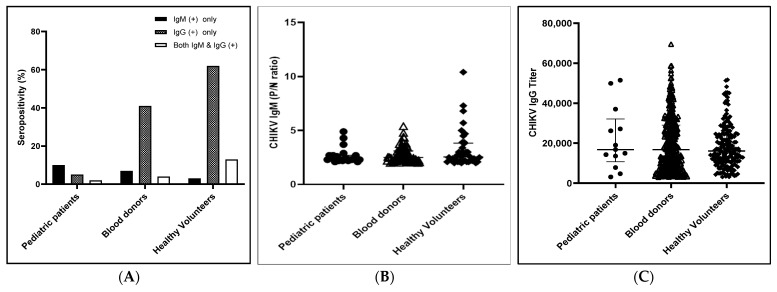
(**A**) Seropositivity rate (anti-CHIKV IgM and/or anti-CHIKV IgG), (**B**) anti-CHIKV IgM only, and (**C**) anti-CHIKV IgG only positivity in different health statuses.

During the study period, positive tests were returned for 139 study participants (12.4%) for anti-CHIKV IgM Ab and 500 (44.3%) for anti-CHIKV IgG Ab (Figure 2A). Among the 196 dengue-suspected pediatric patients, 19 (9.7%) were positive for the anti-CHIKV IgM Ab only, 10 (5.1%) were positive for the anti-CHIKV IgG Ab only, and 3 (1.5%) were positive for both. The positivity rates for the 691 blood donors and 237 healthy volunteers were as follows: anti-CHIKV IgM Ab only, 49 (7.1%) and 8 (3.4%); anti-CHIKV IgG Ab only, 281 (40.7%) and 146 (61.6%); and the combination of the two, 30 (4.3%) and 30 (12.7%), respectively. The seropositivity rates (anti-CHIKV IgM and/or anti-CHIKV IgG Ab positive rate) against CHIKV were 32/196 (16.3%) for dengue-suspected pediatric patients, 360/691 (52.1%) for blood donors, and 184/237 (77.6%) for healthy volunteers. These results show that the seropositivity rate was the lowest among the dengue-suspected patient group in the Mandalay Region and the highest among the healthy volunteers in the Yangon Region. The P/N ratios of the anti-CHIKV IgM Ab were compared among the study populations and no statistical difference was found (*p*-value > 0.05) (Figure 2B). Similarly, the anti-CHIKV IgG Ab titers were compared among the study populations, and no statistical difference was identified (Figure 2C).

### 3.4. CHIKV Neutralizing Antibody Levels among the Different Populations

The participants who tested positive for either the anti-CHIKV IgM or anti-CHIKV IgG Ab were assessed for CHIKV-neutralizing antibodies (NAb) and the percentages of neutralization test positive cases among ELSA positive cases were shown in Table 2. Only 80% of IgM Ab only positive, 96.3% of IgG Ab only positive, and 100% of both IgM and IgG Ab positive by ELISA tests were confirmed CHIKV infection by neutralization test. Neutralizing antibodies with dilutions greater than 1:10 were detected in 31/196 (18.9%) of the dengue-suspected pediatric patients and 309/691 (44.7%) of the blood donors (26.4%) from the Mandalay Region, as well as 180/237 (46.8%) of the healthy volunteers from the Yangon Region. The highest NAb levels against CHIKV consisted of a 1280 titer among dengue-suspected pediatric patients and blood donors and a 2560 titer among healthy volunteers. The proportions of NAbs according to age group among the different study populations are described in Table 3, which shows that Nab levels against CHIKV increased with age.

### 3.5. Comparison of Neutralizing Antibody Levels between Anti-CHIKV IgM Only, Anti-CHIKV IgG Only, and Both Positive Cases

**Figure 3:**
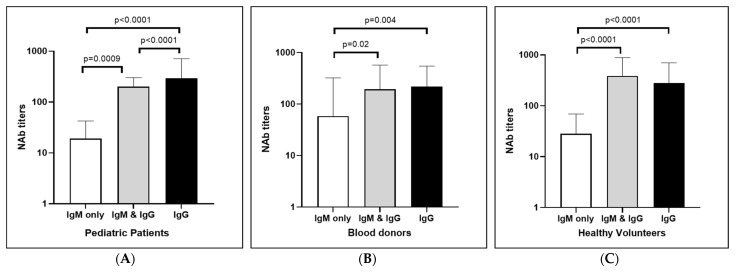
Comparison of neutralizing Ab levels of anti-CHIKV IgM only, anti-CHIKV IgG only, and both positive cases among (**A**) pediatric patients (**B**) blood donors, and (**C**) healthy volunteers.

The mean neutralizing antibody titer was compared among the positive cases for anti-CHIKV IgM only, anti-CHIKV IgG only, and both in the different study populations. The mean neutralizing Ab level in the dengue-suspected pediatric patients was the highest among the IgG Ab only positive cases and was statistically significant when compared with those of the IgM Ab only and the combined Ab-positive patients (Figure 3A). For the blood donors, the mean neutralizing Ab level was similar in the IgG only and the IgM and IgG Ab positive cases. A statistically significant difference was found in the IgG Ab only positive cases when compared to the IgM Ab only positive cases Figure 3B). The mean neutralizing Ab level in the healthy volunteers was the highest in the combined IgM and IgG Ab positive cases and was significantly different from those in the IgM Ab only positive cases (Figure 3C).

## 4. Discussion

DENV, Zika Virus (ZIKV), and CHIKV are endemic to Myanmar and present with similar clinical manifestations [9]. No surveillance has been performed for CHIKV infections; therefore, estimates of the overall burden of the disease, immune statuses against CHIKV infection among the population, and hospital attendant rates in Myanmar are lacking [7,8]. To introduce a new vaccine into the expanded immunization program, baseline data regarding these factors are crucial [18]. Furthermore, policymakers and national immunization technical advisory groups require this information to ensure appropriate targeting of the population when administering the new vaccines.

In this study, the positivity rates were highest with the molecular test and lowest with the serology tests in the dengue-suspected patients compared to the blood donors and healthy volunteers. As CHIKV IgM Ab appears after 5 days post-infection, diagnostic sensitivity will be low if serological tests are used to detect acute CHIKV infections [24]. However, 2% and 8% of molecular tests were positive in blood donors and asymptomatic healthy volunteers, respectively. This data highlights the benefits of using the molecular test for the diagnosis of acute CHIKV infections and the need to strengthen infection prevention control measures and blood donor screening during CHIKV outbreaks.

Moreover, a weak diagnosis was observed when using serological tests, such as cross-reactivity with other alphaviruses (Ross River virus, O’Nyong Nyong virus, etc.) [10]. Although the first-line serologic tests were confounded by the cross-reactivity with the same serogroup, the neutralization test was the superior method to confirm a CHIKV infection [10]. In this study, the neutralization test was performed to confirm both anti-CHIKV IgM and anti-CHIKV IgG. Furthermore, although anti-CHIKV IgM Ab can persist for up to 18 months, it varies with ethnicity and nationality [15]. However, there is currently no data in relation to the Myanmar population. Therefore, if serological tests are the only method used for diagnosis, the results should be interpreted cautiously [10].

The Semliki Forest serocomplex to which CHIKV belongs could conceivably cross-react the serological test results. For example, the Getah virus was isolated from mosquitoes in the Myanmar–China border area and it can infect the Myanmar population and can make the results cross-reactive [25]. However, there was no report on the Getah virus infection inside Myanmar. Among seropositive cases, 80% of IgM only positive and 96.4% of IgG only positive cases were confirmed for CHIKV infection. Among the dengue-suspected pediatric patients, 35.3% were only DENV-infected, 14.9% were only CHIKV-infected, and 29.4% were coinfected with DENV and CHIKV in previous studies [7,8]. The rest could be other kinds of infections and we could not check for other causes of infection in this study. The primer used to detect chikungunya virus in this study had no cross-reactivity with ZIKV, all four serotypes of DENV, West Nile virus, Kunjin Virus, enterovirus 71, echo virus, and other alpha viruses such as Ross River virus as described in the previous studies [8,26].

In contrast, serology tests were used for surveillance or for determining the actual burden of the outbreak, because asymptomatic cases could not be detected by passive surveillance, and only a minimum number of cases are identified using that method. Most asymptomatic cases were detected by active surveillance to determine the actual magnitude of the disease [12]. Approximately 10% to 15% of CHIKV-infected people present as asymptomatic [27]. Therefore, serological tests could be used to understand the burden of this disease. In this study, 11.4% of blood donors showed anti-CHIKV IgM Ab despite the lack of symptoms over a three-month period. Similarly, 16.0% of healthy volunteers tested positive for anti-CHIKV IgM, yet they were asymptomatic. Therefore, serology tests could be used to identify infected cases and explore the burden of this disease.

A CHIKV outbreak started in March 2019 in the southern part of Myanmar, near the Thailand border, and then expanded gradually to the central parts of the country. Therefore, the outbreak affected the more Southern Yangon Region earlier than the Central Mandalay Region. One study of CHIKV infections among blood donors in the Mandalay Region found that the positivity rate was highest in August and September [7]. Therefore, the positivity rate for molecular tests was expected to be lower in samples from Yangon Region than those in the Mandalay Region during this period. However, the positivity rate using the serological test was higher in the Yangon Region, and this was used to explore the burden of the infection in the Yangon Region during the outbreak and confirm the role of serological tests in assessing the infectious disease landscape.

A previous study described that the highest seroprevalence rate of 37.3% occurred in 2018 when compared to 2013 and 2015 [6]. The seropositivity rate in 2018 was 47.9% among healthy volunteers from the Yangon Region and 26.2% among blood donors in the Mandalay Region [6]. In 2019, the seropositivity rate among blood donors and healthy volunteers increased to 52.1% and 77.6%, respectively. Based on one systemic review and metanalysis which was conducted on the studies published between 2001 and 2020, among eight studies conducted only with children worldwide, the pooled seroprevalence was 7% (95% CI 0–23), Seropositive cases were not identified in Tunisia [28]. The lowest seroprevalence was 0.2% in French Polynesia [29] and the highest was 53.3% in Kenya [30]. Among thirty-nine studies conducted on all ages of the population, the seropositivity rate of CHIKV was 30%. The lowest seroprevalence identified was 0.8% in Fiji [31] and the highest was 95.4% in Laos [32,33]. Moreover, in one seroprevalence study in Thailand in 2014, 30.9% of the Thai population had antibodies against CHIKV [34].

In this study, the IgM Ab only positivity rate was highest, and the IgG Ab positivity rate was low in the younger age group. This represented their first infection; therefore, they had no previous exposure or immunity against the virus, although there was no mortality among the study participants. The older age group showed high seropositivity and neutralizing Ab positive rates, which increased with age. These individuals would have been exposed in previous years; therefore, the current infection would indicate a second exposure or repeat infection due to the genotype changes in the virus. However, this study did not explore whether these were first or repeat infections. In this study, the neutralizing Ab levels were highest among IgG Ab positive cases, while low or absent neutralizing Abs were observed in IgM Ab only positive cases as the neutralizing Ab is lower at the subacute phase and gradually increases. IgM Ab only positive cases signify the early phase of infection when IgG Ab had not yet appeared. IgM plays in a complementary manner with the early IgG [35]. The median time for detection of IgG Ab was 10 days. Therefore, the neutralizing Ab levels were not detected.

As the limitations of the study, this study was the combination of three cohort studies such as annual dengue surveillance among dengue-suspected pediatric patients, and arbovirus screening among blood donors and healthy volunteers at study hospitals. To identify the seroprevalence rate, which was confirmed by a gold standard neutralization test, dengue-suspected pediatric patients were enrolled for the prevalence rate of the symptomatic CHIKV infection, and the blood donors and healthy volunteer adult populations were enrolled for asymptomatic CHIKV infection rate. However, symptomatic and asymptomatic populations were not included in all age groups.

In the present study, the seropositivity rate of CHIKV infection was markedly increased during an outbreak year. This study explored the burden of CHIKV infection during an outbreak year in Myanmar. The younger age group showed a lower rate of seropositivity which could be a lower rate of protective immunity. Thus, this study provides the necessary information about the burden of CHIKV infection among children and explores the affected age group which needs protective immunity against the virus. There are many factors to consider for introducing a new vaccine such as disease magnitude, existence and effective prevention strategy, the capacity of the immunization program, underlying health system, safety of the vaccines, etc. [36]. This study explored the burden of the disease for the stakeholders to consider for adding of CHIKV vaccine in the population at the Expanded Programme on Immunization when the vaccines are available in the future.

## Figures and Tables

**Table 1:** Demographic characteristics of the study population.

Variable	Overall Number (%)	Male (%)	Female (%)
**Age(years)**			
≤5	70 (6.2)	43 (61.4)	27 (38.6)
5–15	126 (11.2)	63 (50.0)	63 (50.0)
16–45	872 (77.6)	635 (73.0)	237 (27.0)
≥46	56 (5.0)	39 (69.6)	17 (30.3)
Total	1124 (100)	780 (69.4)	344 (30.6)
**Healthy Status**			
Dengue suspected patients	196 (17.4)	106 (54.1)	90 (45.9)
Blood donors	691(61.5)	508 (73.5)	183 (26.5)
Healthy volunteers	237 (21.1)	166 (70.0)	71 (30.0)
Total	1124 (100)	780 (69.4)	344 (30.6)
**Region**			
Mandalay	887 (78.9)	614 (69.2)	273 (30.8)
Yangon	237 (21.1)	166 (70.0)	71 (30.0)
Total	1124 (100)	780 (69.4)	344 (30.6)

**Table 2:** Percentages of positive ELISA results confirmed by neutralization tests.

	IgM Ab Only (+) (n)	Confirmation Test (n, %) *	IgG Ab Only (+) (n)	Confirmation Test (n, %) *	IgM and IgG Ab (+) (n)	Confirmation Test (n, %) *
Dengue suspected patients	19	17/19 (89.5%)	10	10/10 (100%)	3	3/3 (100%)
Blood donors	49	39/48 ** (81.2%)	281	270/281 (96.1%)	30	30/30 (100%)
Healthy volunteers	8	4/8 (50%)	146	146/146 (100%)	30	30/30 (100%)
Total	76	60/75 (80%)	437	426/437 (96.3%)	63	63/63 (100%)

* Ab positive by ELISA tests confirmed by neutralization test was shown in number (n) and percentages (%). ** Not enough serum sample for doing neutralization test.

**Table 3:** Age distribution of Chikungunya virus neutralizing Ab among the study population.

Age (Years)	Neutralization Titer (FRNT_50_)									Positive/Tested (%)
	10	20	40	80	160	320	640	1280	2560	
**Patients**										
1–3	2	3	0	0	1	0	1	1	0	8/49 (16.3)
4–6	4	0	1	1	0	0	1	0	0	7/60 (11.6)
7–9	3	2	0	1	0	2	0	0	0	8/66 (12.1)
10–12	1	0	0	2	4	1	0	0	0	8/21 (38.0)
**Blood donors**										
18–25	3	2	4	8	15	46	21	3	0	102/277 (36.8)
26–35	3	1	5	14	33	32	14	2	0	104/234 (44.4)
36–45	1	2	7	13	17	19	5	6	0	70/133 (52.6)
46–55	0	0	4	5	7	10	6	1	0	33/47 (70.2)
**Healthy volunteers**										
18–25	1	0	1	8	21	21	19	11	0	82/96 (85.4)
26–35	0	1	1	5	16	20	14	6	1	64/94 (68.0)
36–45	0	0	4	7	2	6	4	2	0	25/38 (65.7)
46–55	0	0	1	1	2	3	2	0	0	9/9 (100)

## Data Availability

The datasets generated and/or analyzed during the current study are available in the manuscript.
